# Outcomes of post-cardiac surgery patients with persistent hyperlactatemia in the intensive care unit: a matched cohort study

**DOI:** 10.1186/s13019-016-0411-5

**Published:** 2016-02-24

**Authors:** Nicole T. J. J. Mak, Sameena Iqbal, Benoit de Varennes, Kosar Khwaja

**Affiliations:** Division of General Surgery, Vancouver General Hospital, 950 West 10th Avenue, Vancouver, Canada; Department of Nephrology, McGill University Health Centre, Montréal, Canada; Division of Cardiothoracic Surgery, McGill University Health Centre, Montréal, Canada; Departments of Surgery and Critical Care Medicine, McGill University Health Centre, 1650 Cedar Ave. L9.411, Montréal, QC H3G 1A4 Canada

**Keywords:** Lactate, Surgery, Cardiac, Surgical intensive care, Acidosis, Lactic, Postoperative care

## Abstract

**Background:**

Higher morbidity and mortality rates are seen amongst patients presenting with hyperlactatemia in the postoperative period. The purpose of this study was to determine the relationship between persistent elevations in lactate and poor ICU outcome in post-cardiac surgery patients.

**Methods:**

This was a retrospective matched cohort analysis of cardiac surgery patients undergoing bypass and/or valve surgery in a university hospital centre. Selection criteria were: cardiac bypass and/or valve surgery; admission to the ICU for > 24 h postoperatively; and peak lactate ≥ 3.0 mmol/L. Hyperlactatemic patients were matched to 2 normolactatemic patients. Multivariable conditional logistic regression was used to determine predictors of hyperlactatemia and mortality.

**Results:**

Four hundred sixty-nine post-cardiac surgery patients were admitted to the ICU for > 24 h. 144 of these patients had an arterial blood lactate ≥ 3.0 mmol/L. Amongst the mortalities, 78.9 % presented with hyperlactatemia. Independent risk factors predictive of a lactate ≥3.0 mmol/L were preoperative IABP insertion (RR 2.8, CI 1.1–7.2) and postoperative acute kidney injury (RR 3.2, CI 2.1–5.4). Patients whose lactate concentrations continued to increase >30 h postoperatively were more likely to die (RR 8.44 CI 2.50–28.53).

**Conclusions:**

The persistence of hyperlactatemia is a more important determinant of postoperative outcome than the absolute value of the peak lactate concentration. A simple postoperative lactate washout does not sufficiently explain this lactate accumulation. Mortality is proposed to be secondary to a state of ongoing hypoperfusion.

## Background

A product of anaerobic metabolism, lactate is a biomarker and indicator for tissue hypoperfusion and oxygen debt [1, 2]. In anaerobic metabolism, pyruvate is converted to lactate so that, in conditions of hypoxia, energy may continue to be derived from glucose. Pathological causes of lactic acidosis include those conditions causing tissue hypoxia: pulmonary disease leading to low P_O2_; circulatory shock with decreased oxygen delivery; and decreased hemoglobin and oxygen carrying capacity [3, 4]. Lactic acidosis secondary to hypoxia or hypoperfusion is known as “type A” lactate acidosis. A rise in lactate may also be of the “B” type, which is due to a lack of lactate clearance by the liver and kidneys.

Lactate concentrations are measured regularly in the intensive care unit (ICU) and elevated blood lactate is a common finding post-cardiac surgery. In the critically ill patient, a normal blood lactate is less than 2 mmol/L. High postoperative serum lactate levels have been previously associated with an increased risk of postoperative mortality and poor outcome in both adult and pediatric cardiac surgery populations [2–10]. Lactate is also a biomarker and potential prognostic tool in the acute care setting. In adult surgical patients, greater mortality rates are seen amongst patients in whom lactate levels normalize more slowly. Non-normalizing lactate levels have been associated with a 100 % postoperative mortality rate [5].

In this matched cohort study, we describe postoperative outcome in a population of hyperlactatemic cardiac surgery patients. An analysis of the relationship between hyperlactatemia, time to peak lactate concentrations and postoperative mortality was performed. The predictors of elevated lactate were determined and the value of serial lactate measurements evaluated.

## Methods

### Study population and matched controls

**Fig. 1:**
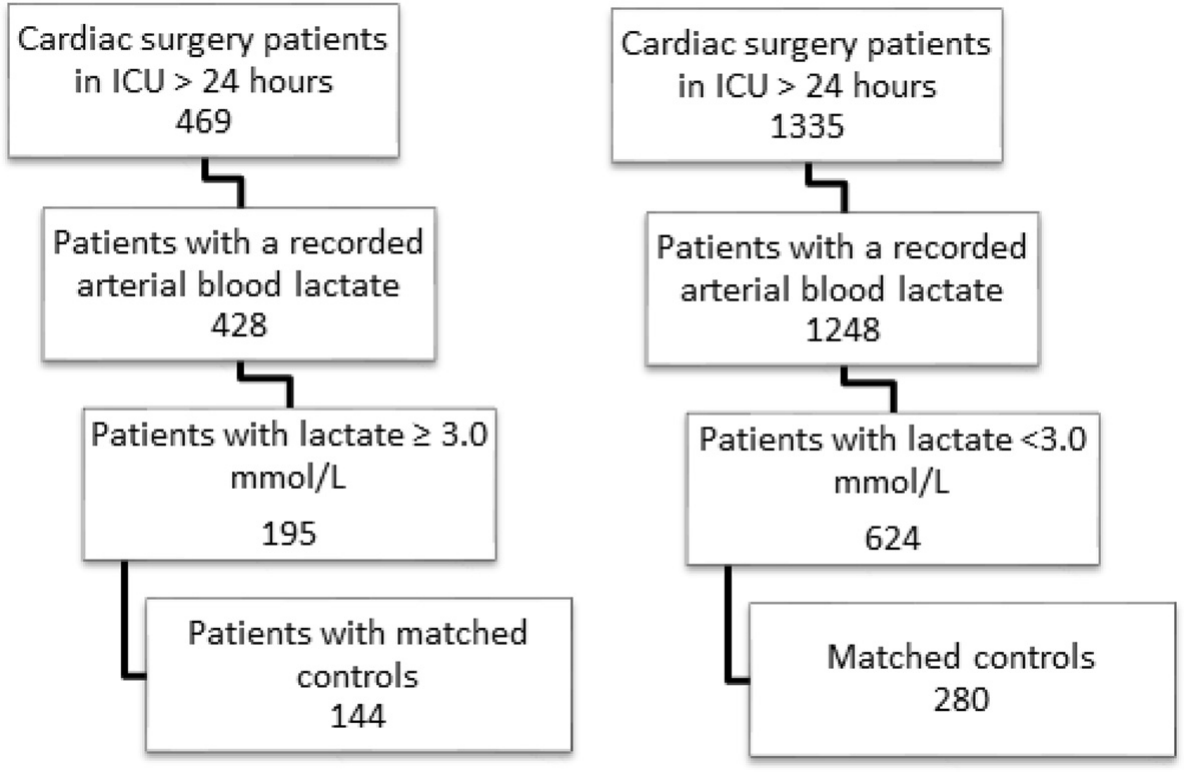
Selection criteria of cases (a) and controls (b) for the matched cohort study

A retrospective chart review was conducted on patients undergoing cardiac surgery at the Royal Victoria Hospital (Montreal, Quebec) from January 2011 to December 2011, inclusive. Inclusion criteria were: 1) elective or emergent coronary artery bypass and/or valve surgery, 2) admission to the ICU for postoperative care > 24 h in duration, 3) peak arterial blood lactate ≥ 3.0 mmol/L (Fig. 1). Each hyperlactatemic patient was matched to 2 control patients selected from cardiac surgery patients whose operation occurred between January 2010 and December 2012. Cases and controls were matched for sex, age, surgery type, Parsonnet score (a risk estimation score of patient pre-operative factors including diabetes, left ventricular ejection fraction, re-operative status, and dialysis-dependence) and 2 preoperative comorbidities associated with mortality risk—renal failure and peripheral vascular disease. Patients without matched controls were excluded from analysis where appropriate.

### Data collection

Preoperative data including comorbidities and risk stratification scores were obtained from the hospital’s cardiac surgery database. Postoperative data was collected from the intensive care unit database and the hospital’s clinical information system database. Additionally, patients presenting with lactate ≥ 3.0 mmol/L in the ICU underwent a chart review. Postoperative data collected was limited to events occurring during the ICU stay. Mortality was defined as death during the cardiac surgery hospital stay. Serial postoperative lactate values for each patient with arterial blood lactate values ≥ 3.0 mmol/L were trended to determine the time to the peak lactate value. This was defined as the number of hours elapsed between the surgery end-time to the time of peak sample collection. Preoperative baseline serum creatinine concentrations were recorded to determine the occurrence of new acute kidney injury using the Acute Kidney Injury Network (AKIN) stage 1 criteria. Lastly, diagnostic interventions performed in the ICU such as CT, MRI, echocardiogram, or ultrasound were noted.

### Statistical methods

Statistical analyses were performed using the SAS System (SAS 9.3 Institute, Cary, NC). Patient characteristics were summarized using proportions, medians, and ranges as appropriate. The data was analysed in two parts. First, a cohort study of the patients with lactate ≥3.0 mmol/L was conducted to determine outcomes. This was followed by a matched cohort study to assess risk factors for hyperlactatemia and mortality.

For the cohort study, student’s t-tests or Wilcoxon’s rank-sum and Kruskal-Wallis tests were used to compare clinical variables of survivors and non-survivors. Variables with univariate significance were entered into the Cox Proportional Hazard regression model which assessed the effect of time to the development of abnormally high serum lactate levels while controlling for possible confounders. A categorical variable was created for the 90^th^ percentile for time to peak lactate concentrations (30 h). Kaplan Meier curves were created to depict the survival curves for those reaching peak lactate before and after 30 h.

In the matched cohort analysis, Wilcoxon’s rank-sum and Kruskal-Wallis tests were used to compare clinical variables between normolactatemic and hyperlactatemic patients as well as between survivors and non-survivors. The individually matched cases and controls were analyzed using conditional logistic regression through the Cox Proportional Hazard model with strata. Variables with univariate significance were entered into the model and predictors for in-hospital mortality were determined. Generalized estimating equations for binary data were used to assess the association of elevated serum lactate levels with postoperative intra-aortic balloon pump (IABP).

Data are presented as the following, as appropriate: number (proportion in percentage), median (range), hazard ratio (95 % CI). A *p*-value < 0.05 was considered significant.

This study was approved by the McGill University Health Centre ethics review board. No patient consent was required.

## Results

### Patient demographics

Postoperative lactate ≥ 3.0 mmol/L was observed in 195 cases out of the 428 patients reviewed. The procedures performed in the patient population included 279 coronary artery bypass grafts (CABG), 29 aortic valve repairs/replacements (AVR), 23 mitral valve repairs/replacements (MVR), 45 CABG + AVR, 7 CABG + MVR, and 4 MVR + tricuspid valve repair/replacement. The matched groups had similar preoperative characteristics for the variables for which they were matched. Baseline demographics and preoperative comorbidities of cases and controls are found in Table 1. Data was non-normally distributed and therefore the median was used to describe and compare cases with controls. Prevalence of preoperative cardiogenic shock and IABP insertion was higher in patients with postoperative peak lactate ≥3.0 mmol/L. The matched controls had a higher frequency of chronic obstructive pulmonary disease and diabetes mellitus.

**Table 1:** Comparison of demographics and perioperative course of cases and controls

	Cases (*n* = 144)	Controls (*n* = 280)	*p*-value
Preoperative characteristics			
Gender (male)	93 (64.6 %)	181 (64.6 %)	1.00
Age (years)	70 (43–89)	70 (42–89)	0.81
Parsonnet score	18.3 (0–49.0)	16.5 (0–52.5)	0.28
Renal failure^a^	22 (15.3 %)	29 (10.4 %)	0.16
Severe peripheral vascular disease	13 (9 %)	17 (6.1 %)	0.32
Cardiogenic shock^b^	5 (3.5 %)	1 (0.4 %)	0.02
Preoperative intra-aortic balloon pump	16 (11.1 %)	10 (3.6 %)	0.004
Chronic obstructive pulmonary disease^c^	10 (6.9 %)	48 (17.1 %)	0.004
Diabetes^d^	38 (26.4 %)	103 (36.8 %)	0.04
Postoperative characteristics			
Peak lactate (mmol/L)	4.6 (3.0–18.0)	2.1 (0.8–2.9)	<0.0001
Peak serum creatinine level (mmol/L)	127 (66–840)	100 (39–1013)	<0.0001
Intra-aortic balloon pump	29 (20.1 %)	14 (5 %)	<0.0001
Computed tomography chest /abdomen	4 (2.8 %)	0	0.01
Return to operating room	22 (15.3 %)	5 (1.8 %)	<0.0001
Cardiogenic shock	9 (6.3 %)	2 (0.7 %)	0.002
Post-operative cardiac hemorrhage	9 (6.3 %)	4 (1.4 %)	0.01
Acute kidney injury (AKIN 1)	68 (50.4 %)	73 (26.8 %)	<0.0001
Ischemic bowel	1 (0.7 %)	0	0.34
Length of ICU admission (days)	3 (1–96)	1 (1–29)	<0.0001
ICU readmission	12 (8.3 %)	13 (4.7 %)	0.13
Mechanical ventilation (days)	0.89 (0.14–75.5)	0.50 (0.09–11.9)	<0.0001
Mortality	15 (10.4 %)	4 (1.4 %)	<0.0001

Cases presented with peak lactate ≥3.0 mmol/L in the postoperative ICU admission while controls had a peak lactate <3.0 mmol/L. Data is expressed as: number of patients (frequency in percentage) or median (range)

^a^Acute or chronic renal failure

^b^With urinary output <10 cc/hr

^c^Chronic obstructive pulmonary disease on medical treatment

^d^Diabetes on insulin or oral hypoglycemic agents

### Postoperative outcomes

A summary of postoperative complications is presented in Table 1. The median peak postoperative arterial blood lactate amongst the cases was 4.6 mmol/L. That of the matched controls was 2.1 mmol/L. Over 15 % of cases returned to the operating room (OR) for further intervention: 8 for hemorrhage/re-exploration, 4 for pericardial effusion, 2 for perforated bowel, 2 for bowel ischemia/obstruction, 2 for peripheral ischemia and 1 for left ventricular assist device. By contrast, only 1.8 % of controls returned to the OR for hemorrhage/re-exploration. The use of IABP was more frequently employed in those with hyperlactatemia (p < 0.0001). A significantly higher occurrence of complications such as cardiogenic shock (6.3 %, *p* = 0.002), cardiac hemorrhage (6.3 %, *p* = 0.01), and acute kidney injury (50.4 %, *p* < 0.0001) were also observed. Renal dysfunction was further characterized by comparing serum creatinine levels. Preoperative baseline serum creatinine levels were similar between both groups but peak postoperative creatinine was found to be higher in those presenting with lactate >3.0 mM. No significant difference was found with respect to the usage of angiography but more CT scans of the chest and/or abdomen were conducted for the hyperlactatemic patients.

Among the cases and controls, length of ICU stay, time on mechanical ventilation and hospital mortality were measured (Table 1). With respect to these measures, hyperlactatemic patients had poorer outcomes, having a longer ICU stay (3 vs. 1 days, *p* < 0.0001), greater time on mechanical ventilation (0.89 vs 0.50 days, *p* < 0.0001), and higher hospital mortality (*p* < 0.0001). The mortality rate amongst hyperlactatemic patients was 10.4 % compared to 1.4 % in the controls. No differences were found in the length of hospital admission or the frequency of ICU readmission.

A comparison between survivors and non-survivors was conducted in the cohort as well as in the matched-control analyses. Comparative analysis revealed that, among the cases of hyperlactatemia, non-survivors had a mean Parsonnet score of 31.5, compared to a score of 16.75 in survivors (Table 2). Non-survivors were more often patients with pre-existing renal failure, chronic obstructive pulmonary disease (COPD), hypertension and heart failure. Emergent surgery, longer cardiopulmonary bypass (CPB) time and requirement for postoperative IABP were also observed to be significantly increased in non-survivors. The mean CPB time amongst those who died was 153 min compared to 110 min in those that survived (*p* = 0.01). Death was associated with a higher peak lactate (10.2 vs 4.4 mmol/L, *p* = 0.0002) and a delayed time to peak lactate. The 90^th^ percentile for time to peak lactate was 30 h. A significantly greater proportion of non-survivors surpassed the 90^th^ percentile: in 64.7 % of mortalities, lactate peak was reached at >30 h (*p* < 0.0001). On average, hyperlactatemic non-survivors attained their peak lactate in 37.6 h compared to 7.5 h in survivors (*p* < 0.0001). Again, differences were noted with respect to renal function. Acute kidney injury was more frequent in non-survivors. Peak creatinine and percentage rise in postoperative creatinine were also greater.

**Table 2:** Comparison of perioperative characteristics of mortality and survival amongst hyperlactatemic patients

	Non-survivors (*n* = 17)	Survivors (*n* = 127)	*p*-value
Pre-operative characteristics			
Gender (male)	9 (52.9 %)	84 (66.1 %)	0.29
Age (years)	75 (56–87)	70 (43–89)	0.12
Parsonnet score	31.5 (17.0–49.0)	16.8 (0–46.5)	<0.0001
Parsonnet score greater than 31	20 (15.8 %)	9 (52.9 %)	0.001
Operation status (emergency)	3 (17.7 %)	14 (11.5 %)	0.03
Surgery type			0.36
Dialysis	3 (17.7 %)	3 (2.4 %)	0.02
Chronic Obstructive Pulmonary Disease	4 (23.5 %)	6 (4.7 %)	0.02
Congestive Heart Failure	11 (64.7 %)	50 (39.4 %)	0.05
Post-operative characteristics			
Cardiopulmonary Bypass time (min)	153 (83–214)	110 (25–302)	0.01
Post-operative Intra-aortic Balloon Pump	8 (47.1 %)	21 (16.5 %)	0.003
Time to peak lactate >30 h	11 (64.7 %)	3 (2.4 %)	<0.0001
Time to peak lactate (hours)	37.6 (1.3–385)	7.5 (0.25–755.5)	<0.0001
Peak arterial lactate (mmol/L)	10.2 (2.4–18)	4.4 (1.7–13.1)	0.0002
Total Mechanical Ventilation Days	3.14 (0–76.5)	0.89 (0.14–44.1)	0.0001
Acute kidney injury	16 (100 %)	64 (51.6 %)	0.0002
Peak creatinine (μmol/L)	309 (107–738)	119 (66–840)	<0.0001
Postoperative rise in creatinine (%)	30 (−80 to 254)	164 (31 to 660)	<0.0001

Cases presented with peak lactate ≥3.0 mmol/L in the postoperative ICU admission. Data is expressed as: number of patients (frequency in percentage) or median (range)

Comparative analysis of non-survivors and survivors in the matched-control population showed that preoperative renal failure, severe peripheral vascular disease and reoperation were associated with mortality while age and congestive heart failure approached significance (Table 3). The majority of patients who died presented with hyperlactatemia (78.9 %) and the 28-day mortality amongst hyperlactatemic patients was 5.6 % (8/144 patients). Mortality in the study population was associated with a higher incidence of postoperative surgical interventions, IABP and complications such as cardiogenic shock, cardiac hemorrhage, and renal dysfunction.

**Table 3:** Comparison of survival and non-survival amongst cases and controls: demographics and perioperative characteristics

	Non-survivors (*n* = 19)	Survivors (*n* = 405)	*p*-value
Preoperative characteristics			
Gender (male)	11 (57.9 %)	263 (64.9 %)	0.62
Age (years)	75 (56–87)	70 (42–89)	0.05
Parsonnet score	30.5 (16.5–49)	16.5 (0–52.5)	<0.0001
Congestive heart failure	12 (63.2 %)	170 (41.9 %)	0.06
Renal failure^a^	9 (47.4 %)	42 (10.4 %)	<0.0001
Severe peripheral vascular disease	5 (26.3 %)	25 (6.2 %)	0.007
Reoperation	3 (15.8 %)	8 (2 %)	0.005
Chronic obstructive pulmonary disease^b^	7 (36.8 %)	51 (12.6 %)	.008
Postoperative characteristics			
Peak arterial lactate ≥3.0 mmol/L	15 (78.9 %)	129 (31.8 %)	<0.0001
Peak creatinine μmol/L	127 (66–840)	100 (39–1013)	<0.0001
Intra-aortic balloon pump	6 (31.6 %)	37 (9.2 %)	0.008
CT chest /abdomen	2 (10.5 %)	2 (0.5 %)	0.01
Return to OR	5 (26.3 %)	22 (5.4 %)	0.005
Cardiogenic shock	5 (26.3 %)	6 (1.5 %)	<0.0001
Post-operative cardiac hemorrhage	2 (10.5 %)	11 (2.7 %)	0.01
Acute kidney injury (AKIN 1)	16 (84.2 %)	125 (32.2 %)	<0.0001
Non-obstructive ileus	1 (5.5 %)	2 (0.5 %)	0.13
Ischemic bowel	1 (5.5 %)	0	0.05

Data is expressed as: number of patients (frequency in percentage) or median (range)

^a^Acute or chronic renal failure

^b^COPD on medical treatment

### Predictors of hyperlactatemia and mortality

Conditional logistic regression was used to determine predictors of hyperlactatemia and mortality. Two factors predictive of a peak postoperative lactate ≥3.0 mmol/L were found: insertion of preoperative IABP and postoperative acute kidney injury. Adjusted hazard ratios are presented in Table 4. Patients requiring preoperative IABP insertion were 2.8 times more likely to develop hyperlactatemia (95 % CI 1.1–7.2). Acute kidney injury was associated with a hazard ratio of 3.2 (95 % CI 2.1–5.4).

**Table 4:** Risk factors for lactate ≥3.0 mmol/L during the postoperative ICU admission

Variable	Unadjusted OR (95 % CI)	Adjusted OR (95 % CI)
Diabetes status	0.53 (0.33–0.86)	0.47 (0.28–0.77)
Preoperative intra-aortic balloon pump	3.0 (1.2–7.3)	2.8 (1.1–7.2)
Acute kidney injury (AKIN 1)	3.1 (2.0–4.7)	3.2 (2.1–5.1)

*n* = 424

**Fig. 2:**
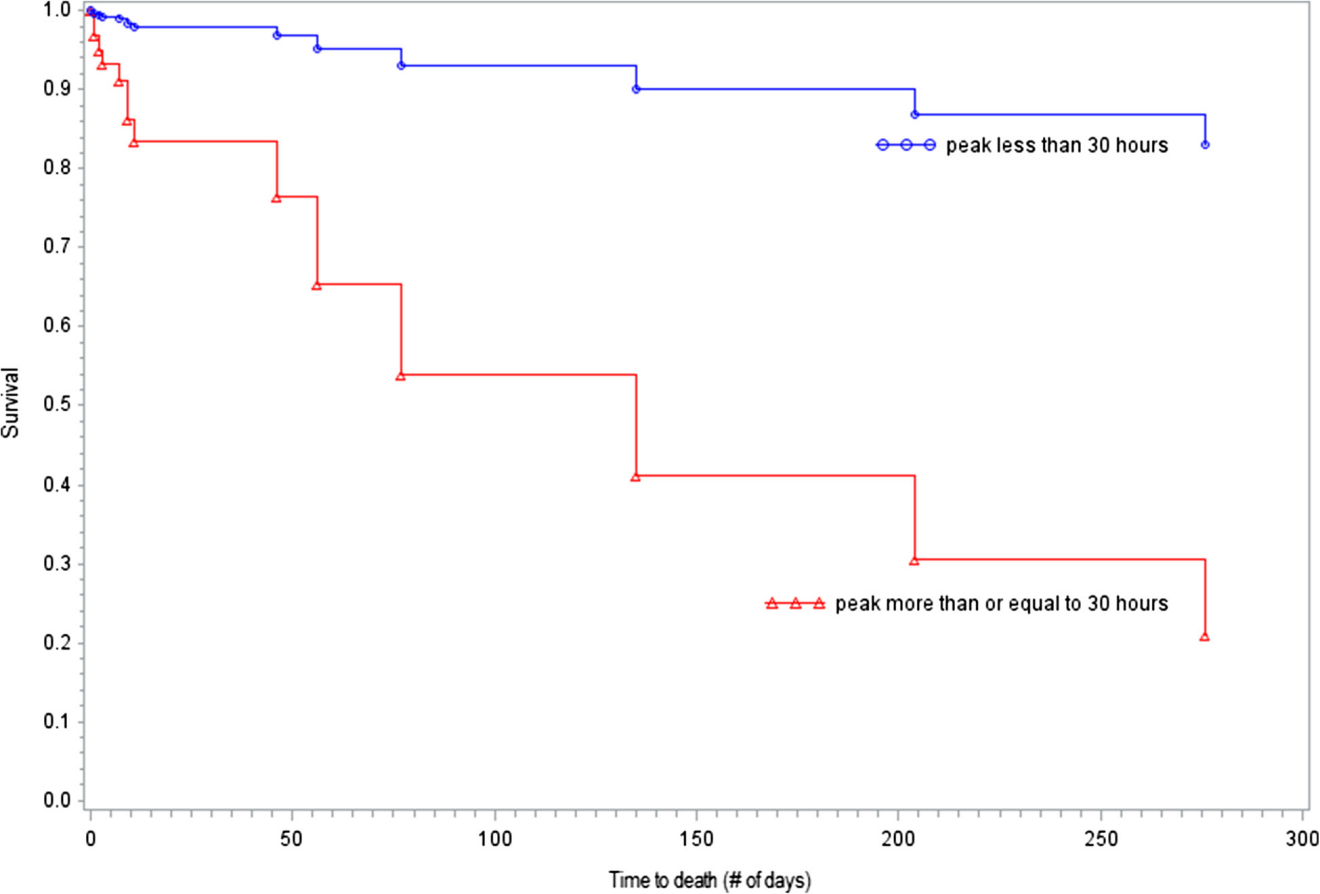
Survival curve for cases of lactate ≥ 3.0 mmol/L showing survival probability for patients attaining peak lactate in ≥ 30 h (triangles) and those attaining their peak lactate in < 30 h (circles)

Despite the association between hyperlactatemia and mortality on univariate analysis, the logistic regression model conducted did not find lactate ≥3.0 mmol/L to be a predictor of death. However, when analysing hyperlactatemic patients as a cohort, calculated hazard ratios showed that a delayed peak in lactate was strongly associated with death (Table 5). Patients who exceeded the 90^th^ percentile for time to peak lactate were 8.44 (95 % CI 2.50–28.53) times more likely to die. Survival curves illustrate that overall survival outcomes were better in those who had a shorter duration of lactate rise (Fig. 2). Other risk factors for mortality were percentage increase in serum creatinine and Parsonnet score.

**Table 5:** Adjusted and unadjusted hazard ratios for mortality in patients with peak postoperative lactate ≥3.0 mmol/L

	Adjusted HR (95 % CI)	Unadjusted HR (95 % CI)
Time to peak lactate ≥ 30 h	8.44 (2.50–28.53)	7.13 (2.17–23.45)
Percent rise in serum creatinine	1.72 (1.15–2.58)	1.40 (1.03–1.90)
Parsonnet score > 31	5.21 (1.07–25.44)	0.80 (0.24–2.65)

*n* = 128

## Discussion

The relationship between hyperlactatemia and poor outcome has been established in adult and pediatric populations. Mortality risk has been associated with both intraoperative and postoperative hyperlactatemia [2–17]. Lactate levels between 2.0 and 4.4 mmol/L have been reported to be associated with increased postoperative morbidity and mortality. Debate exists in the literature regarding which lactate measurement is most indicative of poor outcome. Some have measured lactate concentrations under CPB or peak postoperative lactate while others have found that lactate levels upon ICU admission were predictive of outcome [5, 7–13, 15–19]. Other studies in medical and surgical critical care have reported that better outcomes are observed in the presence of effective lactate clearance [6, 13, 19–23]. In sum, different parameters have been used to characterize the relationship between lactate and poor outcome. Furthermore, evidence exists to suggest that survivors may be in a metabolic state more favorable for early resolution of the hyperlactatemic state. It may be that, in critically ill patients with multi-organ dysfunction, duration of lactic acidosis has greater predictive value than that of initial lactate values at first presentation.

Lactate concentration is determined by both the production and clearance of lactate. An elevated lactate may be secondary to a previous state of anaerobic metabolism that has not yet been cleared. Therefore, the actual metabolic state of the patient may not be determined by a sole reliance on a lactate concentration. Given that hepatic and renal clearance is often impaired in the critically ill, it is reasonable to instead observe serial lactate measurements. In this study, lactate was followed until the peak concentration was reached. That is, measurements were followed so long as there was evidence of a continued imbalance in the production and clearance of lactate. It is expected that time to peak lactate is a better reflection of ongoing active metabolic dysfunction in the critically ill. One other study in the literature by Ranucci et al. is known to have employed a similar strategy. Theirs was a study of intraoperative anaerobic metabolism in patients on cardiopulmonary bypass [17].

The results of this study suggest that a single event of lactate ≥ 3.0 mmol/L is not a reliable predictor of mortality in post-cardiac surgery patients [24]. Instead a strong relationship was drawn between a late peaking lactate and mortality. A peak in lactate at >30 h postoperatively was a highly significant risk factor for death in our population. This risk was independent of the absolute concentration of lactate in the blood, CPB time, Parsonnet score and use of postoperative IABP. Two other risk factors with a significant mortality risk were postoperative rise in creatinine and the Parsonnet score, a risk stratification score previously studied on patients at our institution [25, 26]. These findings bring clarity to the approach to interpretation of serial lactate measurements in the post-cardiac surgery patient population.

Both cardiogenic and non-cardiogenic causes for hyperlactatemia exist in our population. The multiple logistic regression models performed here for hyperlactatemia suggest that kidney dysfunction and requirement for insertion of a preoperative IABP are associated with increased risk of postoperative hyperlactatemia. The pathophysiology of the hyperlactatemic state is primarily one of hypoperfusion, resulting in anaerobic metabolism and lactic acid production. Meregalli et al. argued that circulatory dysfunction is present even in critically ill surgical patients without evidence of shock. It can be inferred that the presence of hyperlactatemia indicates “occult hypoperfusion” in certain patients, thus explaining their increased mortality [23]. Interestingly, in our study, acute kidney injury in the postoperative period, defined by the 2007 Acute Kidney Injury Network stage 1 criteria, is highly correlated with hyperlactatemia. From this, we learn that hyperlactatemia is not only a result of decreased oxygen delivery, but that it is also affected by end organ dysfunction. Interpretation of lactate trends therefore should take into account such non-cardiogenic factors.

In the cardiac surgery population specifically, perfusion deficits are created while on CPB. As core temperature is increased and CPB is weaned, perfusion of organs becomes inadequate for demand, resulting in lactic acidosis [27]. In a prospective study, length of time on CPB has been previously suggested to increase the likelihood of developing a postoperative lactatemia ≥ 5.0 mmol/L [28]. While the perfusion deficits developed during CPB are undoubtedly contributing to the cardiac surgery patient’s anaerobic state in the postoperative period, our results show that CPB time does not have an independent effect of significance on the development of a postoperative hyperlactatemia ≥ 3.0 mmol/L. This lack of detection of a measurable effect of CPB time on lactate elevation may be due to our lower threshold value for hyperlactatemia.

The lactate produced intraoperatively while on CPB continues to be detected in the serum postoperatively as it is cleared by the kidneys and liver. In addition to this postoperative “washout phenomenon”, perfusion deficits may persist into the recovery period, leading to lactate persistence. We do observe that this development of late hyperlactatemia is associated with higher mortality risk. The continued lactate production post-CPB is not sufficiently explained by a post-CPB washout phenomenon. A short duration of lactate accumulation and an early peak in serum lactate—in other words, a washout—likely engender less morbidity and mortality because of the absence of ongoing perfusion deficits. We therefore deduce that a hyperlactatemia of significance is the result of a state of ongoing hypoperfusion, manifesting as a longer duration of lactate accumulation and a greater rise in serum creatinine.

There are limitations to this study. This retrospective review included a heterogeneous study population with different cardiac pathologies and procedures. Data on the use of inotropes, which remain possible confounders in this study, were not collected. Only patients with an ICU stay greater than 24 h were included with the rationale that the hyperlactatemia in these patients was of clinical significance. A bias in the cohort analysis may have resulted from this selection criteria. Note that in the matched cohort analysis, both the cohort and corresponding controls had an ICU admission >24 h.

To summarise the findings of this study, we highlight the concept that the duration of hyperlactatemia is a more important risk factor than the lactate concentration itself. We propose that persistent elevations in lactate, for which time to peak lactate is a surrogate measurement, are due to a state of ongoing hypoperfusion, leading to a significant increase in mortality risk. This is in contrast to an early lactate rise which represents a washout phenomenon.

## Conclusions

Cardiac surgery patients presenting with late peaking blood lactate >30 h post-operatively are at increased risk for poor ICU outcome and mortality. Lactate persistence is a better measurement of prognosis than peak lactate in post-CPB patients. Patients under a state of persistent hypoperfusion, as opposed to a simple post-CPB lactate washout, are at risk for an increased frequency of major postoperative complications and mortality.
